# Developing Workforce Capacity in Public Health Informatics: Core Competencies and Curriculum Design

**DOI:** 10.3389/fpubh.2018.00124

**Published:** 2018-05-02

**Authors:** Douglas R. Wholey, Martin LaVenture, Sripriya Rajamani, Rob Kreiger, Craig Hedberg, Cynthia Kenyon

**Affiliations:** ^1^Health Policy and Management, School of Public Health, University of Minnesota, Minneapolis, MN, United States; ^2^Office of Health Information Technology, Minnesota Department of Health, St. Paul, MN, United States; ^3^Courage Kenny Rehabilitation Institute, AllinaHealth, Minneapolis, MN, United States; ^4^Environmental Health Sciences, School of Public Health, University of Minnesota, Minneapolis, MN, United States; ^5^Minnesota Department of Health, St Paul, MN, United States

**Keywords:** public health informatics, public health practice, public health workforce, systems analysis, systems design

## Abstract

We describe a master’s level public health informatics (PHI) curriculum to support workforce development. Public health decision-making requires intensive information management to organize responses to health threats and develop effective health education and promotion. PHI competencies prepare the public health workforce to design and implement these information systems. The objective for a Master’s and Certificate in PHI is to prepare public health informaticians with the competencies to work collaboratively with colleagues in public health and other health professions to design and develop information systems that support population health improvement. The PHI competencies are drawn from computer, information, and organizational sciences. A curriculum is proposed to deliver the competencies and result of a pilot PHI program is presented. Since the public health workforce needs to use information technology effectively to improve population health, it is essential for public health academic institutions to develop and implement PHI workforce training programs.

## Introduction

With the increasing use of electronic data collection and storage, there is an increasing focus on information and knowledge management systems (1). This results in public health workforce needs for public health informatics (PHI) professionals skilled in designing and implementing these systems. PHI professionals are those who “work in practice, research, or academia and whose primary work function is to use informatics to improve the health of populations” (2). Recent studies highlight the need for informatics training in the public health workforce (3, 4). Similarly, the Council on Education for Public Health (CEPH) includes informatics as a foundational competency for accreditation of public health programs (5, 6). We build on the literature for developing informatics’ savvy public health departments (7–10) and PHI core competency descriptions (11, 12) to design a curriculum for a Master’s in Public Health (MPH) and certificate in PHI. The proposed curriculum differs from many existing health informatics programs by emphasizing the public health informatician’s architectural role as a systems analyst linking public health users and information technology specialists by guiding the analysis and design of information systems. The focus is on: (a) problem definition for public health information systems (PHInfSys); (b) analyzing, designing, and implementing effective PHInfSys, such as surveillance, community health assessment, and population health management systems (11, 13), (c) encoding, collecting, curating, storing, retrieving, and analyzing data to create information, (c) assuring system and data governance, management, integration, confidentiality, and security, (e) creating and managing information technologies, and (f) collaborating in and leading inter-disciplinary and cross-cutting teams.

The foundation of public health is information, collected across a variety of sources with the goal of generating and disseminating new knowledge and instituting actions to improve the public’s health. The need for public health education programs to be informed by current public health needs and employment opportunities has been long noted (14), as has the importance of establishing academic-practice links with public health agencies and training students in practice situations. In 2001, the need for PHI training was noted in an American Medical Informatics Association (AMIA) conference on developing a PHI agenda (15). In 2012, AMIA revisited and updated these recommendations (16). Both reports covered technical topics, such as information architecture and standards, governance and policy, including confidentiality and privacy, and workforce training. These needs are addressed by the PHI training program we describe.

The 2012 AMIA reported noted that the “The public health value chain is composed of business processes and use cases that describe the flow of data and information. Business processes describe how data, information, and knowledge are used by creating a framework that relates work activities to domains of public health function” (16). PHI is a foundation for modernizing public health value chains, including public health department business processes (17), public health surveillance (18, 19), public health emergency response, population health science (20), population health management (21), learning health systems (22), and public health research. PHI is an essential specialization that bridges the digital gap public health agencies face given growing expectations of service and preparedness. PHI integrates knowledge and skills from the information sciences (computer, information, organizational, and systems sciences) with public health expertise and “includes the conceptualization, design, development, deployment, refinement, maintenance, and evaluation of communication, surveillance, and information systems relevant to public health” (23).

While PHI shares some core skills with other health informatics domains, such as bioinformatics or clinical and nursing informatics, it is distinct in necessitating integration of core informatics skills with public health systems, such as surveillance systems, community health assessment, disease/condition registries, and prevention programs in an overall framework of population health science. Public health informaticians (PHInf) differ from information technologists (ITs) because “The focus of IT is to implement and operate information systems (hardware and software) that meet programmatic needs. In contrast, PHInf have a strategic and systems view of how information systems and technology can impact public health, such as how information systems can support public health decision-making. Unlike IT specialists, PHInf work within the larger context of how information systems function within the political, cultural, economic, and social environment and evaluate their impact within the broad sphere of public health. Thus, PHInf are in the unique position to understand how information systems can improve the practice and science of public health while contributing to the evidence-based practice of public health informatics” (11).

Our paper focuses on training PHInf through MPH and certificate programs to provide them a foundation for a PHI professional career. The professional role is one of four related PHI roles identified by the PHI Institute, the Association of State and Territorial Health Officials, and the National Association of County and City Health Officials (NACCHO): executive; manager; professional; and clinical (24). The curriculum design highlighted here focuses on the professional role because it is a frontline position that is the foundation for a career path through management to executive leadership (25).

Our paper describes program design objectives and assumptions, design challenges, and core competencies. This overview provides context and prioritization for the program design.

## PHI Program Design Objectives and Assumptions

The objective of PHI training is to educate PHInf with public health and PHI knowledge and skills that enable them to design and implement effective PHInfSys and have a successful PHI career. The design assumes that PHInf require competencies in (a) public health core competencies (26), functions, and systems [e.g., disease and environmental surveillance (19), population health management (21), and community health assessment (21, 27, 28)], (b) design thinking and systems analysis (29–31), (c) computer, information, organizational, and systems sciences (23, 32), (d) evaluating information systems effectiveness (33), and (e) teamwork and project management.

The core public health functions are assurance, assessment, and advocacy, which are used to maximize population health (34). The PHI program design focuses on understanding how information systems achieve these functions rather than having a sub-goal focus on technological, computer science, or data mining aspects of PHI. Because PHI focuses on designing PHInfSys it has a foundation in design thinking (29–31). Design thinking focuses on identifying problems as gaps in population health that information systems can help reduce by determining the root causes of those gaps and designing and implementing information systems to reduce population health gaps. Systems analysts are the architects who bridge the gap between users and technologies by understanding and translating user needs to system specifications and collaborating with technologists to implement those systems. The goal of systems analysis is to minimize the frequent disconnects between information system development and the usefulness of the resulting information systems (35, 36) by avoiding simply automating existing inefficient information pathways.

To address these disconnects, effective systems analysis requires that PHInf are “well grounded in the fundamentals of organization theory, decision-making, teamwork and leadership, and research methods as well as current and emerging information systems technologies” (37). PHInf also need the ability to design, implement, and evaluate information systems in multiple contexts, using approaches such as realistic evaluation and context-mechanism-outcome configurations (CMOc) (33).

Finally, PHInf need strong teamwork and project management skills because “PHI is cross-cutting. … Informaticians see the ‘big picture’ and ‘connects the dots’ across all of the other fields related to public health” (13). Because information systems span diverse domains, the public health informatician requires the knowledge and skills to collaborate with diverse clients, lead systems analysis and development, and coordinate the diverse specialists implementing information systems, data use and privacy agreements, data and vocabulary standards, evaluation, training, and more.

In sum, PHInf require competencies in (a) public health core concepts and systems, (b) systems thinking and systems analysis, (c) computer, information, organizational, and systems sciences, (d) evaluating information systems effectiveness, and (e) the ability to work in and lead teams. These competencies provide the foundation for successfully progressing from being a PHI professional to PHI leadership positions.

## PHI Program Design Challenges

Public health informatics requires many different competencies. This can result in a laundry list of competencies that are not feasible to implement in a credit-constrained curriculum. Implementing a constrained educational program requires competency prioritization and trade-offs. There are three major challenges.

(1) *Content*: PHI requires competencies in distinct domains—public health, health informatics, computer, information, and organizational sciences (2, 12, 32). These include required core competencies for public health practice (38). Health informatics and computer science focus on technical competencies. Information, systems, and organizational science competencies support solving population health problems because they provide systems analysis and design competencies needed to: (1) understand and translate user needs into system requirements and (2) create designs that can be implemented by ITs. Computer science competencies, such as modular decomposition, data structures, algorithms, programming languages, database theories, and system architectures are necessary to provide PHInf the knowledge and skills to communicate and collaborate effectively with ITs implementing PHInfSys.
(2) *Curricular independence vs integration*: Siloed, independent courses maximize covering competency breadth, but rely on students to integrate across competencies. Courses integrating competencies develop the skills to apply informatics in public health. Examples of integrating competencies include using population health management and surveillance examples in technical courses, such as systems analysis, database, or health information exchange.
(3) *Declarative vs procedural competencies*: Declarative competencies are familiarity with analysis tools, such as requirements analysis, business process modeling, use cases, data flow diagrams, and entity relationship diagrams. Procedural competencies are being able to develop requirements, business process models, use cases, data flow diagrams, and entity relationship diagrams. Training in procedural competencies requires experiential practicums in which students apply declarative competencies. Increasing the emphasis on procedural competencies limits the breadth of declarative competencies that can be covered. The PHI design focuses on developing strong procedural competencies in system analysis because it is the core competency used to design and implement effective PHInf.

In sum, design challenges include decisions about content, integration (independent/integrated), and competency type (declarative/procedural) tradeoffs.

The proposed PHI design focuses on public health core competencies and organization/systems/information sciences related to analyzing, designing, implementing, and disseminating effective information systems in public health. Integrating competencies across courses maximizes the ability to apply skills in public health settings. For declarative and procedural learning, experiential practicums assure the ability to apply declarative knowledge. Elective credits support students in developing deeper skills in areas they are interested in, such as data management, analytics/data mining, GIS, visualization/communication, health information exchange (39), leading community health information exchange, or surveillance systems (19).

## PHI Competencies

A PHI competency is a measurable and demonstrable knowledge related to the role “of developing innovative applications of technology and systems that address public health priorities by analyzing how information is organized and used and evaluating how this work contributes to the scientific field” (11). The master’s level competencies for PHI professionals build on earlier work (11, 12). Not all competencies listed in earlier work are included for three reasons. First, the selected competencies need to fit into a typical MPH curriculum. While there are many desirable and important competencies, there is limited curriculum time. Second, some competencies reflect work in a practice setting that are not directly measurable (e.g., collaborating with others in program development, offering insights, contributing to decision-making, leading knowledge management). Third, some competencies are more managerial/executive competencies than professional competencies.

### Core Competencies

Core competencies include both public health and PHI. Public health core competencies (6, 26) are addressed through the required MPH curriculum courses. This material is extended by integrating competencies related to public health systems, surveillance systems, population health management, community health assessment, eHealth, program monitoring, and evaluation in the PHI courses. For example, using electronic disease surveillance, population health management, community health assessment, and other public health systems as examples in the systems analysis courses educates students in the use of systems analysis to address common public health systems (17, 40).

Public health informatics competencies focus on systems analysis and data modeling. These include computer science and health informatics competencies related to nomenclature, standards, platforms, information architecture, inter-operability, and systems analysis and data modeling which require integration of material from organization/information systems/systems sciences, public health, and computer sciences. This integration builds the overall competency to design PHInfSys to solve problems in the public health domain. These competencies are noted as central to PHI. For example, “Redesigning Public Health Surveillance in an eHealth World” demonstrates the importance of systems analysis when it points out that “Defining requirements is a critical step in developing or acquiring an information system that will effectively support the work of the organization” (40). Similarly, system selection depends “on the clarity and applicability of the use cases you define, and on determining prior to the demonstration how—based on what criteria—the use-case demonstrations will be judged” (40) as well as business process definitions (40). The description of an informatics savvy health department states that “Information systems managers and staff require competency in creating formal system requirements” (7). Requirements, use cases, and business process definitions are produced by systems analysis and require expertise in the system development life cycles (SDLC), national standards, and public policy related to informatics (e.g., meaningful use) (8). Complementary competencies are those that support systems analysis and data modeling (41), such as standards (e.g., HL7), nomenclature (e.g., ICD, CPT) (42), data flow diagrams, relational database theory, and query languages that inform system analysis and are used during implementation.

As an example, Table 1 illustrates systems analysis competencies for electronic disease surveillance. The goal of the system is to maximize population health by minimizing transmission of communicable diseases, which is done “through early identification, treatment, and resolution of health conditions” (17). Systems analysis requires integrating competency in systems analysis with expertise in the system’s public health domain (e.g., electronic disease surveillance). The first analysis phase defines the problem, its decision-makers, context, and stakeholders. The second phase does a root cause analysis of gaps in population health related to communicable disease surveillance or system productivity and describes functional requirements, what tasks the communicable disease surveillance system supports, and nonfunctional requirements, criteria that the system has to meet. The third phase describes the logical model, the use cases corresponding to each functional requirement, business processes, data flows, and data model. Design and implementation is the final phase and builds on the analysis in the earlier phases to design and implement the information system that supports communicable disease surveillance staff in their activities.

**Table 1:** Systems analysis example for electronic disease surveillance.

Phase	Components	Example
1: problem definition	Problem definition: decision-makers	State disease surveillance staff, local public health disease investigators, information technology specialists at MDH, and in health systems

	Problem definition: goals	Minimize communicable disease incidence and prevalence

	Problem definition: context, stakeholders	When an event is entered into the system, it is used by MDH program staff, epidemiologists, or local health department disease control and prevention staff for case investigation. Users entering into the system include local public health, laboratories infection prevention

	Team charter	Team design—team membership by professional competency that are needed to address the problem (e.g., epidemiologist, clinician, and informaticians), team design (roles, coordination mechanisms), norms, climate (e.g., mutual respect, psychological safety), and leadership

	Problem definition: contingencies	Change in funding streams and budgets to support the electronic disease surveillance system, changes in technology that improve or impede the functionality of the disease surveillance system in relation to other database systems

	Problem definition: preliminary context diagram	Identify physical, political, administrative, and conceptual boundaries, system scope, key information flows, and partners, such as laboratories, clinics, hospitals, CDC, and the public

	Problem definition: problem statement	Disease reporting is necessary to implement interventions, track disease trends, detect outbreaks, and recommend prevention and control measures. The ability to do this in a timely fashion would be enhanced with a centralized electronic disease surveillance system, where there is prompt flow of information between the reporter (lab/clinic) and the investigator (MDH/LPH), rather than separate disease databases that do not support electronic data transmission

	Problem definition: problem scope	Centralize disease reporting into a person centric system that is able to receive electronic lab reports and health care provider reports. In addition, be able to be accessed by multiple different users while maintaining a high level of security.

2: system	Problem statement	Allow for electronic lab reporting directly into the disease surveillance system to facilitate timely notification and follow-up of reportable diseases while conforming to all federal, state, and local regulations addressing health data privacy and protected health information confidentiality

	Root cause analysis	Identify root causes of gaps in maximizing population health associated with communicable diseases by major type of cause (e.g., policies, personnel, information technology, and partnerships)

	Requirements: general	Support public health communicable disease surveillance, encode, store, retrieve communicable disease event reporting from partners (e.g., laboratories, clinics, hospitals, and schools), export data for analysis, transmit reportable disease data to partners (e.g., CDC, other states)

	Requirements: functional requirements	Encode, store, and retrieve communicable disease events (surveillance) Support case management/outbreak investigation Follow-up on communicable disease events resolution Provide decision support for communicable disease management Exchange data with other approved systems (interoperability), such as CDC Export data for analysis using statistical software Support on-boarding of health information exchange partners Demonstrate value of electronic disease surveillance

	Requirements: non-functional requirements	Assure timely, real-time reporting Assure accuracy and validity of reporting Assure both sensitivity, identifying all possible outbreaks, and specificity, minimizing false alarms of reporting Assure capacity to handle high volume outbreaks without wasting capacity User-friendly interface that is consistent throughout the system Ability to manually enter and import disease surveillance data into disease-specific variables in the system Ability to securely store and transfer encrypted data Secure information and functions using roles Support inter-operability with partners (labs, clinics, hospitals, schools, other states, and CDC) Support inter-operability with other surveillance systems (vital records, immunization, environment, and food safety) Support standardized health information exchange (e.g., current versions of HL7) Ensure reliable and timely information access Scalable to respond to increases in diseases monitored and health information exchange increases. Economically sustainable given public health fund constraints.

3: logical Model	Use cases	There should be a detailed use case for each functional requirement showing actors, preconditions, postconditions, data, and detailed actions to perform use case

	Business process models	Detailed business process models using business process modeling notation (BPMN) for the key business processes. The key business processes should show the relationship among use cases in business processes

	Data flow diagrams	Detailed data flow diagrams (DFD) showing information flows from external entities to processes and data stores. The top level DFD is the context diagram showing the key external entities and information flows for the system as a whole. The processes should be consistent with use cases and BPMN. The data stores should be consistent with the data model

	Data dictionary and data model	Detailed data dictionary providing metadata for system data (e.g., type, length, and valid values). The data model should show normalized entity relationship diagrams or equivalent logical data models

4:implementation	System architecture	Verify the system can handle the amount of end users that will be using it. Make sure ample memory is available to avoid system slowdowns. Ensure redundancy between the server and end users is in place in the event of server hardware failures, storage failures, and network connectivity related issues

	System implementation options	Purchase software from vendor and implement using internal staff and vendor staff

	Organizational implementation plans	Implement and maintain database through support from software vendor, internal IT, disease subject matter staff, and informaticians. Establish regular meetings with stakeholders to ensure ongoing alignment between business users needs and database functionality

	Security and confidentiality	Ensure that system requirements meet state requirements for data security, and reportable disease requirements for privacy and confidentiality

	Evaluation plan	Use routine stakeholder meetings to evaluate system alignment with needs of the disease surveillance goals. Ensure stakeholders’ needs and goals are met

	Sustainability and adaptation plan	Maintain funding for desired system enhancements unavailable with routine upgrades of the system. Specifically, have a budget for future upgrades, service, and training

### Declarative and Procedural Competencies

Tables 2 and 3 show the MPH PHI declarative and procedural competencies, which have a strong focus on those competencies also used in systems analysis and data modeling. These competencies reflect the informaticians’ architect role, which focuses on the problem definition and solving skills associated with defining problems, identifying requirements, building logical models, describing core features of information systems (use cases, data flows, data models, business processes), designing implementation alternatives, overseeing implementation, and facilitating user centered design and choice.

**Table 2:** Public health informatics (PHI) declarative competencies.

**Public health**

Public health declarative core competencies as defined by public health professionals Describe core public health information systems, such as population health management, surveillance, and community health needs assessment systems Describe the relationship of PHI to the National Public Health Performance Standards and the core public health functions (assess, assure, advocate) Describe core public health interventions, such as accountability and public reporting, and the mechanisms by which they work Describe population health management and population definitions (e.g., geographic populations, patient populations)

**Health informatics**

Describe conceptualizations of data, medical vocabulary nomenclature, terminologies (e.g., ICD, SNOMED, and CPT), coding and classification, standards (e.g., UML, BPMN), ethics, privacy, security, health systems (e.g., EHRs), computer technologies (e.g., XML), health information exchange (e.g., HL7, DUAs), data quality, and information architectures Describe systems analysis methodology, requirements, use cases, data flow diagrams, business process modeling, data modeling, relational data theory, normalization, entity relationship diagrams, SQL, and data warehouses Technologies and activities to support implementing public health systems, such as interoperability, health information exchange, data use, and business associate agreements Confirm that public health data transfer processes and repositories operate within appropriate standards for security and privacy. This includes compliance review with HIPAA, ASTM, HITSP, IHE, and ISO standards, as well as with state and local requirements

**Systems analysis and development and data modeling**

Describe the systems analyst role in a public health context Describe the systems analyst role in leading systems analysis and design (SAD) and effective team work Describe system development life cycles and their phases (planning, analysis, design, and implementation) Describe a project charter and its categories, describe team organization Describe project context and stakeholder analysis goals and categories Describe requirements specifications and types of requirements specifications (e.g., business, user, functional/non-functional) Describe feasibility analysis and feasibility types (e.g., technical, economic, and organizational) Describe logical models (use cases, business process modeling, data flow diagrams, context diagrams, data modeling/entity relationship diagrams, business rules, and algorithms/pseudocode) Describe implementation alternatives (e.g., system architecture, user interface design, program design, interoperability, and security) Describe implementation strategies (e.g., training, parallel/cutover implementation)

**Evaluation and analysis**

Describe the components of research articles and how to evaluate the components Describe the relationship between concepts, causation, and measurement Describe types of measurement, validity, and reliability and how they relate to research design and implementation Describe nomological networks and measurement reliability and validity Describe the strengths, weaknesses, and uses of qualitative, quantitative, and mixed methods approaches and techniques of data collection and analysis Describe and compare strengths and weaknesses of research designs, such as experimental, quasi-experimental, observational, retrospective, and prospective Describe and compare strengths and weaknesses of traditional research designs and community based participatory research Describe user centered design evaluation methods Describe systematic evaluation methods Describe realistic evaluation using context-mechanism-outcome configurations Describe analytic issues common to health care, such as risk adjustment, sampling, use of operational data affect data quality, and analysis decisions Describe ethical issues in conducting evaluations and research Describe control charts, funnel plots (league tables), and spatial outlier detection and relate their use to public health and describe the data structures required.

**Table 3:** Public health informatics (PHI) procedural competencies.

**Public health**

Procedural public health core competencies as defined by public health professionals “Assess uses and value of different types of data to answer public health questions” (12) “Present PHI information to lay and scientific audiences”(12) “Collate and analyze data to produce intelligence that informs decision-making, planning, implementation, performance monitoring, and evaluation” (46)

**Technical**

“Monitor the use of data security management concepts and principles”(12) “Identify data needs and obtain, verify and organize that data and information”(46) Construct transactions for health information exchange Use structured query language to measure quality, resource use, access, and population health Aid the public health organizations in thinking through and designing testing strategies for new systems

**Systems analysis and development and Data Modeling**

Define information system in terms of solving public/population health problems, develop project charters, lead systems development teams through all project phases with appropriate milestone presentations and reports Analyze the context of an information system, the key stakeholders and their interests, institutional issues, standards and architectures, constraints, resource available, the fit between organizational adaptability and information architecture Elicit, analyze, and report requirements from users using interviews, focus groups, observation, document using tools, such as problem analysis, root cause analysis, and user centered design(12) Conduct and report a feasibility analysis for a proposed system; facilitate the review of the problem statement, charter, requirements, and feasibility analysis for decisions about analysis and implementation Develop and report integrated logical models consisting of integrated (a) problem statements, (b) use cases, (c) data flow and context diagrams, (d) business process models, and (e) data models that are appropriately normalized Document business rules using algorithms/pseudocode Design and evaluate alternative implementation strategies based on the logical models and user centered design principles; design information architectures Oversee, coordinate, and guide the implementation for client organizations “Manage data and information in compliance with policy and protocol”(46) “Assess and manage risks associated with using and sharing data and information, data security, and intellectual property”(46) “Predict future data need and develop data capture methods to obtain it”(46)

**Evaluation**

Evaluate empirical research articles in terms of research design, methodology, and inferences Conduct systematic and realistic evaluations of PHI systems “Assess the usability and user satisfaction of an information system or application, and its utility, including effectiveness and efficiency”(12)

**Professional**

Write and present professional quality analyses; facilitate meetings; communicate with users effectively using user language (not jargon)

The declarative and procedural competencies also include a significant focus on research analysis and evaluation methods because PHInfSys are interventions designed to improve population health by improving quality, reducing costs, or both. Given the intervention aspect, a PHInf should have the competencies to be able to evaluate whether the public health information system accomplished its goals and how the information system functions in different contexts [realistic evaluation (33)]. Effective evaluation requires enough expertise in public health systems, such as disease surveillance (18), quality improvement (43), public reporting, such as control charts, funnel plots (league tables) (44), and spatial outlier detection (45), to be able to assess how well they are implemented as well as their effectiveness. Competencies in assessing measurement validity and reliability are central to measuring population health.

### Electives

Electives allow students to pursue specializations in related public health and PHI competencies such as:

- PHI management: project management, health information management, public health leadership, and leading change for regional and community information systems.
- PHI tools: ePublic health, using the web and mobile apps for health promotion, GIS for public health, health care operations research and analytics, public health decision support systems, eHealth, health information exchange, and surveillance systems in depth.
- PHI evaluation: decision analysis for policy analysis, cost-effective analysis, program evaluation, usability engineering, and realistic evaluation.
- Environmental health systems: Complex systems modeling for population health, infectious diseases, environmental health risk assessment, surveillance of foodborne diseases and food safety hazards.
- Analytics and data mining: Advanced statistical computing, statistical learning and data mining, survival analysis, statistics for surveillance and quality improvement.
- Computer science: Algorithms and data structures, principles and practice of database systems, data marts and warehouse management, NoSQL and alternatives to relational data models.

## Organizing Competencies into Courses

The design addresses siloing vs. integration in two ways (Table 4). First, given that the application of informatics competencies is conditional on public health context, informatics and public health competencies are strongly integrated. Students need the expertise to apply the informatics concepts in the domain they will practice. Second, a practice-based practicum in the introductory systems analysis course and a practice-based capstone systems analysis course integrate practice and academics.

**Table 4:** Public health informatics (PHI) curriculum and competencies.

Courses/year		Health informatics	PHI	Systems analysis	Data governance and curation	Population health management	Evaluation
Introduction to health informatics	1st	X	X	X			

Introduction to PHI	1st		X			X	

Public health systems analysis design and development	1st		X	X	X	X	X

Public health systems analysis design and development practicum	1st			X	X	X	X

Managing electronic health information	1st		X	X	X	X	

Data and information for population health management	1st		X		X	X	X

Advanced PHI applications	2nd		X		X	X	

public health systems analysis design and development capstone	2nd		X	X	X	X	X

Principles of public health research, biostatistics, and epidemiology						X	X

**Competency areas**							

Health informatics: nomenclatures, terminology, transport, syntax, semantics, information architectures, security, privacy, inter-operability, and health information exchange							

Public health informatics: public health information systems, surveillance, community health needs assessment, health information exchange, population health management, and role of public health informatician							

systems analysis: system development life cycles, problem definition, context, stakeholders, charter, scope, requirements, logical model (use cases, data flows, data models, business processes), implementation, and risk analysis							

Data governance and curation: relational database theory, normalization, business rules, pseudo-code/structured English, SQL, population health measurement coding (numerators, denominators), NoSQL, security, and privacy/confidentiality							

Population health management: measuring, analyzing, and managing interventions to improve population health where populations are defined both in terms for health systems, the individuals served by a health system, and public health, the individuals living in a geographic area							

Evaluation: systematic evaluation, realistic evaluation, research design, sensitivity/specificity/positive predictive value, and measurement theory							

As an example, informatics competencies in systems analysis courses (introduction and capstone) are illustrated using PHInfSys. The relational database course (managing electronic health information) uses population health measurement for SQL assignments. Data and information for population health management, which focuses on substantive issues related to population health management uses relational database theory and SQL for assignments in population health management and frames the evaluation of public health interventions using realistic evaluation. In sum, the design emphasizes the integration of systems analysis, database, and evaluation competencies with public health systems and population health management. The objective is to train students in informatics competencies, public health competencies, and the use of informatics skills in public health settings. A separate course addresses competencies that are general and applicable to many areas, such as standards, nomenclature, health information exchange layers, and technical issues.

## Implications for Policy and Practice

This paper proposes a master’s level public health curriculum to support workforce development in PHI competencies. The competencies are similar to those described for public health core competencies (6) and in the Advanced Health Informatics Certification (47) with the goal of preparing PHInf who “Practice health informatics with an operational focus on information and knowledge problems that directly impacts the practice of health care, public health, and personal health” (47). Edward Baker and his colleagues recently made a strong business case for PHI (48) arguing that public health is an information intensive business and that health information systems are transforming the way that information management speeds responses to health threats, promote health, and supports public health decision-making. The foundation of these systems are the essential public health services, a focus on population health, and core informatics competencies, such as system analysis, database theory, and health informatics. The implementation of effective PHInfSys requires individuals with the competencies described in this paper.

This program design was based on Carnegie Mellon’s highly successful information systems program which focus on systems analysis and information technology skills to “design and implement effective solutions to meet organizational and management needs for information and decision support ” (37). The program was implemented in the School of Public Health at the University of Minnesota in the Fall of 2013. The first year courses have been offered, evaluated, and refined five times and the second year courses have been offered and refined four times. Thus far, 19 students have earned PHI certificates signifying completion of PHI core courses; 15 will have completed the MPH in PHI by May 2018. The certificate attracted diverse students, including students from health informatics, pharmacy, environmental health, and epidemiology. Applicants have expressed interest in PHI because they feel that data and information is critical to public health research and practice and they feel that learning informatics and analytics in a public health context will help them to identify opportunities for improving population health. Students in the PHI program have had internships supporting quality improvement and population health management with a safety net clinic network, database administration in health care systems and professional organizations, and supporting standards implementation in electronic disease surveillance systems in state health departments. Graduates of the PHI program have been successful in securing positions across private and public health sectors and some have pursued further degrees in health informatics. Seven of the 10 MPH-PHI graduates are employed in positions related to PHI. Table 5 provides a sample of their job descriptions. The job responsibilities span the spectrum from leading projects in PHI and population health management to offering technical support in data management and research coordination. Future program evaluation should measure the degree to which graduates are engaged in public health related activities, are engaged in PHI related activities, initial placement, and career growth.

**Table 5:** Recent master’s in public health public health informatics graduates position examples.

Position title	Position description
Design lead, Senegal	Lead the analysis, design, and implementation of a care management system to track migrants/refugees and the services they receive in a regional, six country (Algeria, Gambia, Ghana, Mali, Niger, and Senegal) response to the problem of irregular migration and slavery in West Africa. The system will track what service use and will be extended to refer migrants to houses/services along their journey

Director of health information technology and billing, safety net primary care practice	Responsible for IT and all EHR systems (Medical/Dental). Collaborate with all departments to establish the best practice workflows and optimize user experience. Manage reporting and onboard training for all new employees. Used expertise from systems analysis and design to implement a paperless system starting at the front desk with registration forms, insurance card scanning process, and all forms that are needed at any clinic

Systems management supervisor, local health department	Supervise PHI staff; manage PHI systems for all of public health, including a EHR and 2–3 other systems used by the PH department; coordinate department wide technology/informatics plan; ensure implementation of best practices related to electronic data transmission, storage, HIPPA, information security standards, data archiving practices, record management, and other aspects of electronic data management; establish standards for the use and performance of data systems and computer systems; document applications and direct development of work procedures; provide leadership to plan, design, and maintain quality assurance of the department’s data systems

Data analyst, health system	Responsible for data analysis, reporting and data management for various clinical quality and safety applications; analyze, interpret, and report data in order to support decision-making, quality improvements, and meet regulatory requirements; participate in the data governance and application design of the system-wide patient safety application Develop oversight reports of the patient safety application in order to ensure accurate data and appropriate follow up action is taken Develop business requirement documents to translate customer needs into actionable and clearly defined specifications; evaluate data reliability and validity; use control charts and statistical analyses to identify intervention targets; collaborate in workgroups to improve analytics and report usefulness

Research assistant, academic health center	Participate in research of staff roles in health practice to improve population health by limiting unintended consequences; systematically analyze the veracity and integrity of verbal comments in interprofessional work; analyze work roles of staff as they relate to electronic health record workflow and determine implications for work flow and training design; study simulations to assess the effectiveness of electronic health record layout and templates

Identity manager, health information exchange firm	Oversee data reconciliation projects, resource management and allocation, staff development, business development, and budget management; responsible for project strategy, planning, and execution; assure data integrity and data quality solutions for health information exchange related to linking records from disparate systems; collaborate in projects with stakeholders related to maintenance of electronic health records and health information exchange in the acute care and physician settings

There are development and sustainability issues that occur because PHI is a new field. First, program development requires collaborating with faculty and stakeholders to communicate what PHI is and a vision of its value (13). Second, marketing and social media programs that convey PHI’s contribution to new and existing public health students need to be developed. Third, partnerships with stakeholders for course based projects and integrating practice with academics have to be developed. Fourth, PHI program development requires funding commitment from schools of public health until enrollment reaches a break-even point. Finally, PHI faculty who have competency in both public health and informatics need to be developed. Until PHI as a field is more institutionalized, a potential tactic for doing this is recruiting post-doctoral staff from fields within public health (e.g., epidemiology, environmental health) and have them develop PHI competencies while developing PHI related research and/or recruiting post-doctoral staff from related informatics disciplines (49) and have them develop public health competencies while developing PHI related research and teaching. While these barriers appear substantial, PHI should be sustainable because of the growth in informatics related disciplines and because of skilled PHInf being in high demand as valuable team members in both research and practice.

## Conclusion

The effective and efficient collection of quality public health data and information is intensive. It is the foundation of public health functions, such as surveillance, community health assessment, evaluation, and research. PHI is a profession that focuses on the design of governance, analysis, development, and management of information systems that acquire and integrate data that is used in public health functions, such as disease and environmental surveillance, program evaluation, policy analysis, and research. PHI provides the foundation for organizing and collecting data that is used in performing public health functions. PHI is an emerging, distinct, and needed profession in public health that uses knowledge and skills drawn from computer, information, and organizational sciences and public health to develop critical information systems that provide the data and information that other public health professionals use in their work. PHInf provide the expertise in public health, information, and information technology to take advantage of technology advances to improve the PHInf.

This paper proposes a PHI curriculum for schools of public health that can be implemented through MPH and certificate programs. The curriculum provides core competencies in public health and in computer, information, and organizational sciences that are needed by PHInf. The proposed curriculum addresses the challenges of integrating PHI in public health functions, developing declarative knowledge and procedural skills in PHI through a balance of lecture and practicum courses, and allowing the pursuit of specializations. A key focus of the proposed curriculum is its user centered design through systems analysis and systems thinking. This provides PHInf the competencies necessary to lead, design, and implement PHInfSys that take advantage of technology advances.

The proposed program is a starting point. Given the rapidity of change in public health practice, public health research, informatics, and information technology, the curriculum will need to evolve to meet changing needs. This evolution should be guided by partnerships with stakeholders in practice, other public health disciplines, and public health researchers to assure that PHInf are effectively designed and developed to meet the needs of those working to improve population health. This development can be guided by the continued evaluation of PHInf to assure they meet user needs and the continued evaluation of PHI programs to assure they are training the workforce that can lead developments in PHInfSys.

There is a clear need to develop a PHI workforce. It is imperative that public health institutions, both practice and academic, take action and support the development of a strong PHI workforce. This can be achieved through on-the-job training, fellowships, internships, and academic training. The educational program we propose addresses schools of public health, and provides guidelines for implementing PHI training programs. However, support is also needed from public health practitioners to ensure that PHI training is prioritized to meet the evolving demands of the practice environment. With these tools we can continue to develop an effective and relevant workforce that can navigate the changing trends of public health to improve population health.

## Author Contributions

All authors contributed to the conceptualization of this paper. DW, RK, and SR led the curriculum conceptualization and development. ML, SR, and DW led the focus on public health informatics (e.g., the informatics savvy health department, immunization systems), CH, SR, and CK led the focus on applications of PHI in electronic disease surveillance system. DW and ML did the first draft. RK, SR, CH, and CK edited and provided components (e.g., description of electronic disease support systems, contents of systems analysis competencies). All read the final version, edited it, and approved it for submission.

## Conflict of Interest Statement

The authors declare that the research was conducted in the absence of any commercial or financial relationships that could be construed as a potential conflict of interest.
